# Medication by Absorption

**Published:** 1874-12

**Authors:** 


### MEDICATION BY ABSORPTION.

WHEN in Boston the other day our attention was called to a method of surface-medication adopted by Dr. Cooper, of Cambridge, Mass., and with which, we were informed, some remarkable cures had been performed among some neuralgic and rheumatic sufferers. We naturally concluded that the old and well-known principle of "counter-irritation" was involved in Dr. Cooper's method, and were not much inclined to enter upon an investigation of his system. But our friend assured us that the treatment possessed elements of decided novelty, and was, moreover, a genuine success. We accordingly felt it to be our duty to look into it more fully.

We called at the office of the proprietor, and examined the means which he employs, and which he claims to possess great merit and considerable novelty. We were shown pads for the chest and back, united by elastic straps. These pads are cut of the same form as the multitude of "chest-protectors" which have come into extensive use of late. They are made of two thicknesses, one being soft leather, the other flannel. As a protector for the vital organs they are, of course, valuable. Between the flannel and the leather there is quilted the medicating substances. These. as stated to us by Dr. Cooper, are sulphate of potassium, protoxide of hydrogen, bi-borate of soda, protoxide of sodium, and sulphate of alluminum. All are duly commingled and reduced to a fine powder. The moisture and warmth of the body dissolves some particles of these salts as they dust through the flannel, which is held in contact with the skin, and absorption goes on, producing a positive effect upon the animal economy, and supplying some of the elements needed in certain diseased conditions.

This is Dr. Cooper's theory, though, we must confess, that, as a mere theory, it struck us as rather vague and unsatisfactory. The belief has been general that absorption of substances applied to the cuticle occurs only to a very limited extent, and that the true skin must be removed before the constitutional effects of medical agents locally applied are induced. But we have lived too long, and have seen too many startling and successful innovations upon old systems, to be willing to assert that whatever we do not happen to understand must, necessarily, be unscientific and wrong. So we thought it but fair to seek out some of the parties who had used these appliances, and ask them to testify.

We were told that a first-class apothecary in Boston, Mr. T. Metcalf, of 39 Tremont Street, had these goods for sale. We called at his store, but did not find him in. We conversed, however, with an intelligent clerk, Mr. Richards. He said he had been wearing a belt of this kind for a number of months, for relief from a rheumatic affection of the back, and that he secured complete relief by this means. He said that the testimony of nearly every person who used the pad was strongly in favor of it. He spoke of one lady who purchased them in large quantities to give to sufferers. Mr. Richards also alluded to the case of a Mr. John Motley, a prominent citizen of Boston, who had been cured of a very painful rheumatic affection by wearing the pad and belt.

Seeking elsewhere for evidence of the value of the new treatment, we received valuable testimony from a Mr. Kimball, 120 Tremont Street, Boston; C. F. Sherman, a watchman at the Boston and Maine Railroad Depot, residing at East Somerville, Mass.; and of Albert A. Stevens, 26 Fanuel Hall Square. These parties all commend the system in high terms, and seem to think that they cannot do without it in cases of headache, neuralgia, and rheumatism. One gentleman assured us that though he used the appliance only for severe rheumatism between the shoulders, the effect of wearing the pad was to cure a chronic bronchitis from which he had suffered about fifty years.

We went back to Dr. Cooper's store, determined to do him justice. We told him that the testimony was ample, and that we felt bound to so state in public and in private. We were told that the articles could be found at the several stores in New York of the Messrs. Hegeman, who would send them by mail, when desired. Dr. Cooper's place of business is on the corner of Washington and Hanover streets, Boston. The cost of the pads and belts—the latter being for pains in the back—is from $1 to $3, according to size. It is proper to state that the articles are protected by Letters Patent of the United States. While we cannot, as yet, personally vouch for the excellence of this remedial agent, we are bound to say that the honest testimony of those who have fully tested it is of a very convincing character. For full particulars and circulars, Dr. Cooper may be addressed by mail.

